# The microorganisms and metabolome of *Pinus radiata* Pollen

**DOI:** 10.1186/s40793-024-00656-4

**Published:** 2024-12-18

**Authors:** Charlotte Armstrong, Syaliny Ganasamurthy, Kathryn Wigley, Celine Mercier, Steve Wakelin

**Affiliations:** 1 Scion, Christchurch, 8011 New Zealand; 2 Scion, Rotorua, 3010 New Zealand

**Keywords:** Pollen, Microbiome, Gymnosperms, Conifer

## Abstract

**Background:**

Pollen is a crucial source of nutrients and energy for pollinators. It also provides a unique habitat and resource for microbiota. Previous research on the microbiome of pollen has largely focused on angiosperm systems, with limited research into coniferous gymnosperms. This study characterises the pollen microbiome and metabolome associated with one of the world’s most widely grown tree species, *Pinus radiata*. Trees were sampled from locations across Canterbury, New Zealand. Repeated collections were undertaken in 2020 and 2021.

**Results:**

Metabolomic analysis revealed the main compounds present on *P. radiata* pollen to be amino acids (principally proline), and carbohydrates (fructose, glucose, and sucrose). Although phenolic compounds such as ρ-coumaric acid and catechin, and terpenoids such as dehydroabietic acid, were present at low concentrations, their strong bioactive natures mean they may be important in ecological filtering of microbiome communities on pollen. The * P. radiata* pollen microbiome was richer in fungal taxa compared with bacteria, which differs from many angiosperm species. Geographic range and annual variation were evaluated as drivers of microbiome assembly. Neither sampling location (geographic range) nor annual variation significantly influenced the fungal community which exhibited remarkable conservation across samples. However, some bacterial taxa exhibited sensitivity to geographic distances and yearly variations, suggesting a secondary role of these factors for some taxa. A core microbiome was identified in *P. radiata* pollen, characterized by a consistent presence of specific fungal and bacterial taxa across samples. While the dominant phyla, *Proteobacteria* and *Ascomycota*, align with findings from other pollen microbiome studies, unique core members were unidentified at genus level.

**Conclusion:**

This tree species-specific microbiome assembly emphasizes the crucial role of the host plant in shaping the pollen microbiome. These findings contribute to a deeper understanding of pollen microbiomes in gymnosperms, shedding light on the need to look further at their ecological and functional roles.

**Supplementary Information:**

The online version contains supplementary material available at 10.1186/s40793-024-00656-4.

## Background

It is well established that plants harbour a wide diversity of microorganisms in the phyllosphere and other above-and below-ground tissues [82]. Until recently, however, the microbiome associated with plant reproductive organs has been largely overlooked. Yet emerging research is showing that pollen, for example, can be host to diverse communities of bacteria, fungi, viruses, and other microbiota [54]. The discovery that pollen has a microbiome has implications not only for individual plants and their reproductive success [88], but also complex, ecosystem-level outcomes [83].

Studies on the microbiome of pollen have largely focussed on angiosperm systems (i.e. flowering plants) and has been shown to impact ecosystems via influences on pollinator ecology [18]. For example, it has been posited that global decline in bees is linked to the use of agricultural chemicals resulting in dysbiosis of the pollen microbiome that pollinators require and/or have an immune defence against [88]. As pollen is a major dietary resource for bee larvae, the microbial community on pollen is a major factor influencing the health and fitness of these pollinator species [20]. Additionally, links between pollen microbiome and human allergenicity are evident [53]. Bacterial toxins can add to the load of allergens on pollen with outcomes on human health [51, 59]. Thus, from complex and idiosyncratic impacts on structure and functioning of ecosystems through to public health, the implications of the pollen microbiome are profoundly impactful yet vastly understudied.

Pollen represents a vital transport shuttle for movement of microorganisms onto and among plants, and among tissues within individual plants. It facilitates dispersal of microorganisms within macro-ecosystems and transmission across larger geographic ranges [75]. The pollen microbiome is, of course, ideally situated for colonisation of the ovule and seed after pollination [4]. This supplies a direct path for vertical (paternal) microbiome inheritance and coevolution with the host [58, 81, 84]. Similarly, this process provides a pathway for pathogens or other symbionts to enter and impact plant organs grown for productive purposes such as nuts and fruits [11, 21]. Pollen can contain significant amounts of mono- and oligo-saccharides (sugars), nitrogen, protein, micronutrients such as K, S, Cu, Fe, Zn, and other metabolites [16, 28]. During transport in the environment, this may provide resources that sustain epiphytic microbial cells. Conversely, other microorganisms actively parasitise pollen, utilising the material as a resource base per se, and as a source of transported inoculum for decomposition of litter and other debris within the wider ecosystem [36].

Forest plantations are an important source of ecosystem services such as wood and fibre. The fast growth rate of plantation forest trees enables them to supply of 35% of world's wood supply while comprising only 5% of global forest area [25]. Increases in demand for wood and other services, including biofuel, food, and other forest bioproducts can come either at the expense of large natural ecosystems or more intensively managed productive forests [26]. In order to grow the global bioeconomy, increased utilisation of highly productive planted-forests may not only provide raw carbon–neutral resources but avoid biodiversity loss and other ecosystem impacts associated with sourcing materials from natural (e.g. old growth) forest systems.

*Pinus* spp. are extensively used in planted forests globally [24]. When plantation forests are grown as monocultures, these areas of intensive *Pinus* spp. can be responsible for massive pulses of pollen into the ecosystem annually; indeed, this can comprise a key phenological event occurring in forested ecosystems (e.g. [46, 72]). In New Zealand, for example, approximately 90% of the entire planted forests comprise of *Pinus radiata* [29]. This single species covers an extent of 1.6 MHa of land area and, based on the estimates of Fielding [27], may release ~ 300 million kg of pollen each spring (~ 270 kg per ha, depending on site, environment, silvicultural regime, and tree genetics). This constitutes a massive input of carbohydrate, nitrogen, other nutrients, into the environment [34, 36]. It might also facilitate an exchange of microbiomes from the tree to the local and broader surroundings. From a public health perspective, this is certainly nothing to sneeze at. However, the wider ecological significance of such a heavy pulse of pollen and its microbiome into the environment have yet to be investigated.

The purpose of this study is to fill a critical knowledge gap on the occurrence of pollen microbiome of coniferous (gymnosperm) tree species. Conifers are important globally, dominating the composition of boreal forests which comprise 24% of total global tree cover alone. Conifers are also important in temperate forest systems that span Europe, Asia, the Americas, Africa, and Australasia. With a few exceptions, most conifers are needle-leaved evergreen trees; these make up ~ 38% of the trees present globally [49]. Given the focus of (already limited) pollen microbiome studies towards angiosperm spp., this helps addresses a huge gap in our knowledge per se. This work focuses on *Pinus radiata* as it provides a useful model system for tree-microbiome research, and in particular, conifer microbiome research [1–3]. In addition to describing the pine pollen microbiome, we aimed to determine how much of the community is variable versus stable/conserved by sampling trees across a wide geographic range and across two consecutive seasons (years). Finally, given the focus on exploration of a new microbiome habitat, we used a non-targeted metabolomics approach to determine the range of potential metabolites on the surface of *Pinus radiata* pine pollen that may provide resources, or inhibitory molecules, that may support microbial growth or provide a filter for microbiome community composition.

## Methods and materials

### Sample collection

**Fig. 1:**
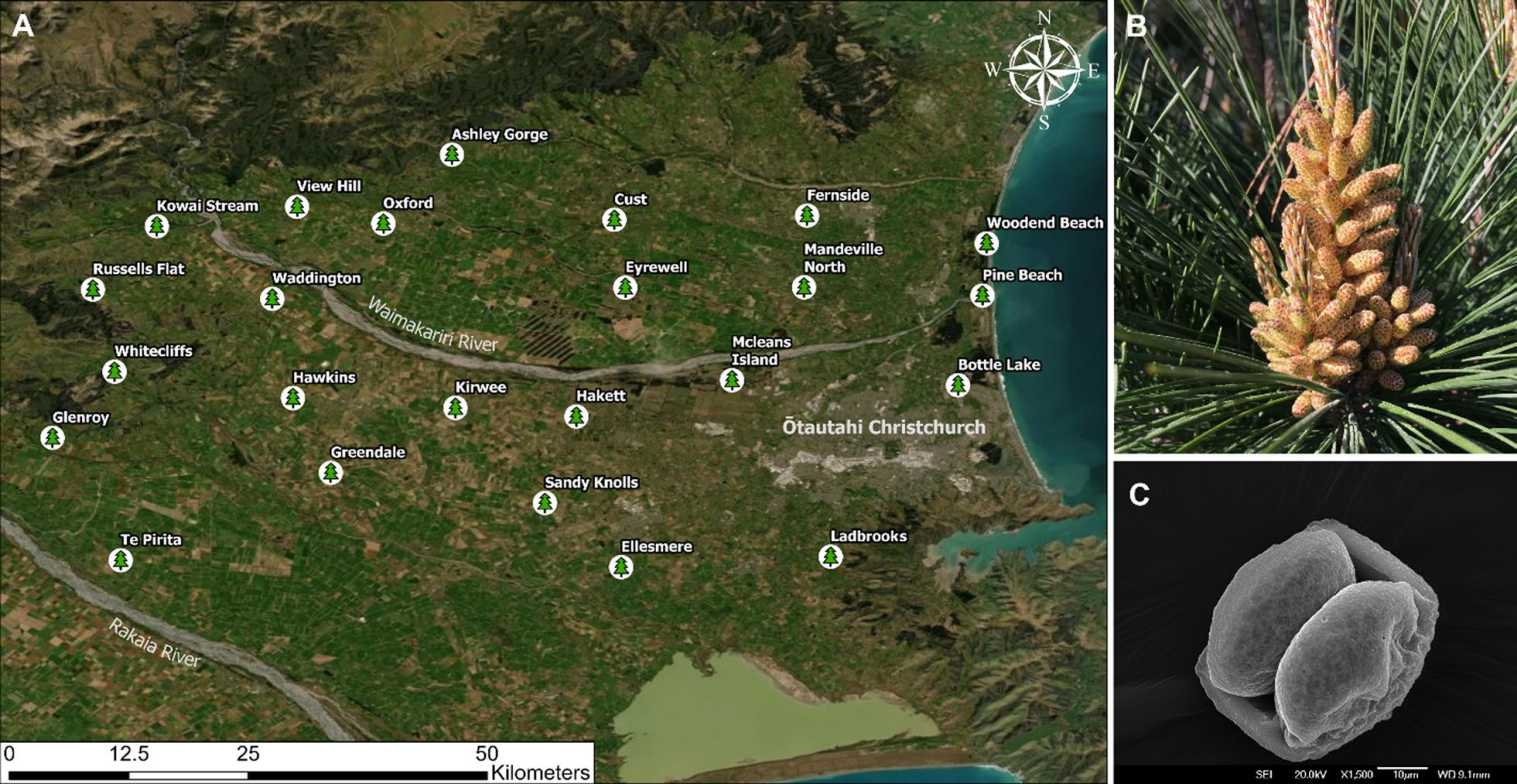
**A** Locations of the 24 *Pinus radiata* trees from which pollen was sampled from. Green pine symbols denote the locations of the trees and the name of the location is next to this symbol. The map is a satellite image of Canterbury based on *Google Earth* imagery for September 2021. **B** A cluster of *P. radiata* catkins (microsporangiate strobili) in September 2021, showing the catkins at pollen-release stage. **C** Scanning electron microscope (SEM) image of a *P. radiata* pollen grain; scale bar 10 µm

Twenty-four *Pinus radiata* trees were randomly selected across the Canterbury region in New Zealand (Fig. 1). This region provides a diverse range of locations ranging from coastal areas, planted and natural forests, recreational parks, agricultural land, rivers, and foothill environments. The selected trees were mature, exhibited good health without observable indications of stress or disease, and had accessible male cones (microsporangiate strobili) at the beginning of spring (September) when they begin to open and release pollen. The male cones of *P. radiata* are often referred to as catkins [64], however these differ to catkins typically associated with angiosperm trees such as birches, alders, willows and others. Unless stated otherwise, we use the term catkin in respect to *Pinus-*type microsporangiate strobili. Pollen from the same trees was sampled in 2020 and again in 2021.

For each tree, a group of catkins clustered along a stem were excised off the branch using sterilised scissors. These were placed in a sterile tube and pollen was removed from the catkins by gentle shaking. The tubes were chilled until returned to the laboratory where they were stored in a − 80 °C freezer until all samples were collected.

Surface features and observation of microbial presence on *P. radiata* pollen was examined under a scanning electron microscope (SEM). Fresh pollen grains were initially sputter-coated with palladium (Emitech K975X coater) before being placed in a JEOL JSM IT-300 variable pressure SEM. Microscopy was conducted at the University of Canterbury Electron Microscopy Centre (Earth and Environmental Sciences Department). Similar to other *Pinaceae* species, *P. radiata* has bisaccate pollen, in which two air sacs protrude from the pollen wall. These sacs facilitate long distance wind dispersal [74].

The metabolome resource available for microorganisms on the surface of *P. radiata* pollen was characterised. Specimen *P. radiata* trees, growing adjacent to a commercial plantation forest at McLeans island (Fig. 1A) were sampled in 2023. At the site, 12 samples of pollen were collected from 4 aspects of 3 trees. Tubes containing pollen were gently rotated in 3:3:2 ACN:IPA:H_2_O (acetonitrile, isopropanol, water) solution to extract polar and non-polar metabolites off the pollen surface. The ACN:IPA:H_2_O extractant was processed by West Coast Metabolomics (California, USA) and compounds were separated and identified using gas chromatography—time-of-flight—mass spectrometry (GC–TOF–MS). Data was provided as normalised peak heights to represent the relative semi-quantification of each compound.

### DNA extraction, library preparation and amplicon sequencing

The pollen was carefully processed to prevent cross-contamination. Due to pollen’s static nature only one sample tube was opened at a time, thorough cleaning of gloves and pipettes in between samples was performed, and sterile filter tips were used. Genomic DNA was extracted from 50–100 mg of pollen using the DNeasy PowerSoil DNA Extraction Kit (Qiagen, Germany). Pollen was transferred to a tissue disruption tube by adding the initial buffer directly to the pollen containing tubes, facilitating easier pipetting and minimizing airborne dispersal. The rest of the DNA extraction protocol was followed as per the manufacturer's recommended instructions. DNA was processed for bacterial and fungal sequencing, targeting the respective 16S rRNA and ITS housekeeping gene regions. The primers used to amplify these genes were based on protocols from the Earth Microbiome Project [10]. The 16S rRNA primers were 515F (5′-GTGYCAGCMGCCGCGGTAA-3′) and 806R (5′-GGACTACNVGGGTWTCTAAT-3′) [62], amplifying the V4-V5 hypervariable region and ITS amplification used primers ITS1f (5′-CTTGGTCATTTAGAGGAAGTAA-3′) [37] and ITS2 (5′-GCTGCGTTCTTCATCGATGC-3′) [85], amplifying the ITS1 region. Primers were barcoded using the Golay 12-mer system, including Illumina adapters. The no-template controls produced no amplification for both 16S and ITS PCRs. Amplicons were cleaned using a magnetic bead PCR clean-up kit (Geneaid, Taiwan), quantified using Quant-iT™ PicoGreen™ dsDNA Assay Kit (Invitrogen, USA), and pooled to an equimolar mix. The 16S and ITS libraries were sequenced using Illumina MiSeq sequencing with 250 bp pair end (PE) read chemistry (16S) and 300 bp PE read chemistry (ITS); this was conducted at the Australian Genome Research Facility (AGRF).

### Bioinformatics and data processing

Raw sequencing data was processed using the DADA2 pipeline outlined for 16S rRNA gene and ITS tag sequences [9]. 16S and ITS reads were demultiplexed using Illumina’s bcl2fastq (Version 2.20) with a barcode mismatch allowance of 1 [38]. ITS Primers were trimmed using cutadapt [55] with default parameters from the DADA2 ITS Workflow (1.8). For filtering, the maximum number of errors allowed were set to maxEE(2,5) for both 16S and ITS, with other standard filtering parameters as per DADA2 workflow. Read length cut-off was set to (250,230) for the forward and reverse reads for the 16S rRNA gene. Chimeric sequences were removed using the removeBimeraDenovo tool provided by DADA2, and taxonomy was assigned to amplicon sequences variants (ASVs) using the assignTaxonomy tool and the RDP reference database (Version 18) for 16S [15] and the UNITE database (Version 8.3) for ITS [42] gene regions. Bacterial and fungal ASV count and taxonomy tables were filtered to remove unassigned sequences, groups other than Bacteria and Archaea (16S specific), singletons, and samples with less than 150 reads. Additional filtering to remove plastid sequences was performed for the 16S rRNA gene dataset.

### Statistical analysis and graphs

Statistical analyses were performed in R version 4.0.5 [66] and PRIMER/PERMANOVA+ (PrimerE Ltd.). A phyloseq object [56] was created in R by merging the ASV count table, taxonomy table, and metadata,this was the base for much of the subsequent data visualisation and statistical analysis. Rarefaction curves were produced with the vegan package [61]. Alpha-diversity was assessed as observed richness of ASV’s [40] and *p* values were computed using a Mann–Whitney test (between two groups). Boxplots were created using GraphPad Prism version 9.4.1 (GraphPad Software, USA).

To identify factors potentially influencing pollen microbiome composition, PERMANOVA modelling [5] was conducted with sampling year and cardinal location (see later). This analysis was conducted using Bray–Curtis calculated distances and performed in PRIMER/PERMANOVA+. Non-metric MDS ordination were generated in R. The metacoder package [30] was used to compute the hierarchical distribution of taxonomic groups in terms of abundance. The clustered heatmap was computed using the pheatmap [41] package in R. The core microbiome plots were generated using the UpsetR [17] and microbiome packages [44]. All plots were generated using ggplot2 [86] unless otherwise stated.

In the initial PERMANOVA testing of main effects, the influence of geographic location on microbiome composition was tested by grouping the samples together based on splitting the sample area into cardinal location quadrants from an approximate centroid. This testing allowed for a ‘cardinal location’ term to be included in the model alongside sampling year. However, this was exploratory in nature and does not provide a consistent means of investigating this variable. In order to identify other underlying microbiome changes with location, geographic distances were calculated and correlated against class-level calculated similarity distance matrices (Bray–Curtis) using Spearman’s-rank correlation. Geographic distances were computed using the Geographic Distance Matrix generator [23] based on sample latitude and longitude input datum.

To partition which individual groups within the community exhibited distance-decay relationships, secondary testing was conducted using the BIO-ENV routine [13, 14] in PRIMER/PERMANOVA+. In this approach, distance matrices (Bray–Curtis) were created individual and combinations of class-group of bacteria and rank-correlations among these and geographic distance was calculated. A permutation-based approach was then used to test for significance of the effects.

Metabolome data were averaged across the 12 samples to obtain a single, representative, *P. radiata* pollen metabolome. The data was classified into superclass groups and the relative quantifications of the compounds were standardised as a percentage of total compounds per sample. Note that this is purely descriptive analysis. No formal testing of location or other effects were tested, rather a typical signature of the pollen metabolome generated.

## Results

### Bacterial and fungal microbiome richness

A total of 9,438,243 and 7,325,595 reads were processed through the DADA2 pipeline for bacteria and fungi respectively (Table S1). At the end of the pipeline remaining reads were accounted for, of which 7,690,837 were from bacteria and 4,551,793 were from fungi (Table S1). Post DADA2, one or more of these routine filtering techniques (i.e. removal of plastid sequences, unassigned groups, Kingdoms other than Bacteria and Archaea (16S Specific) were applied depending on the type of dataset (i.e. Bacteria or Fungi) (Table S2). Filtering resulted in the retrieval of 709 ASVs for bacteria and 2632 ASVs for fungi from 9807 and 4,269,246 total filtered reads respectively (Table S2) which was the basis for all figures.

**Fig. 2:**
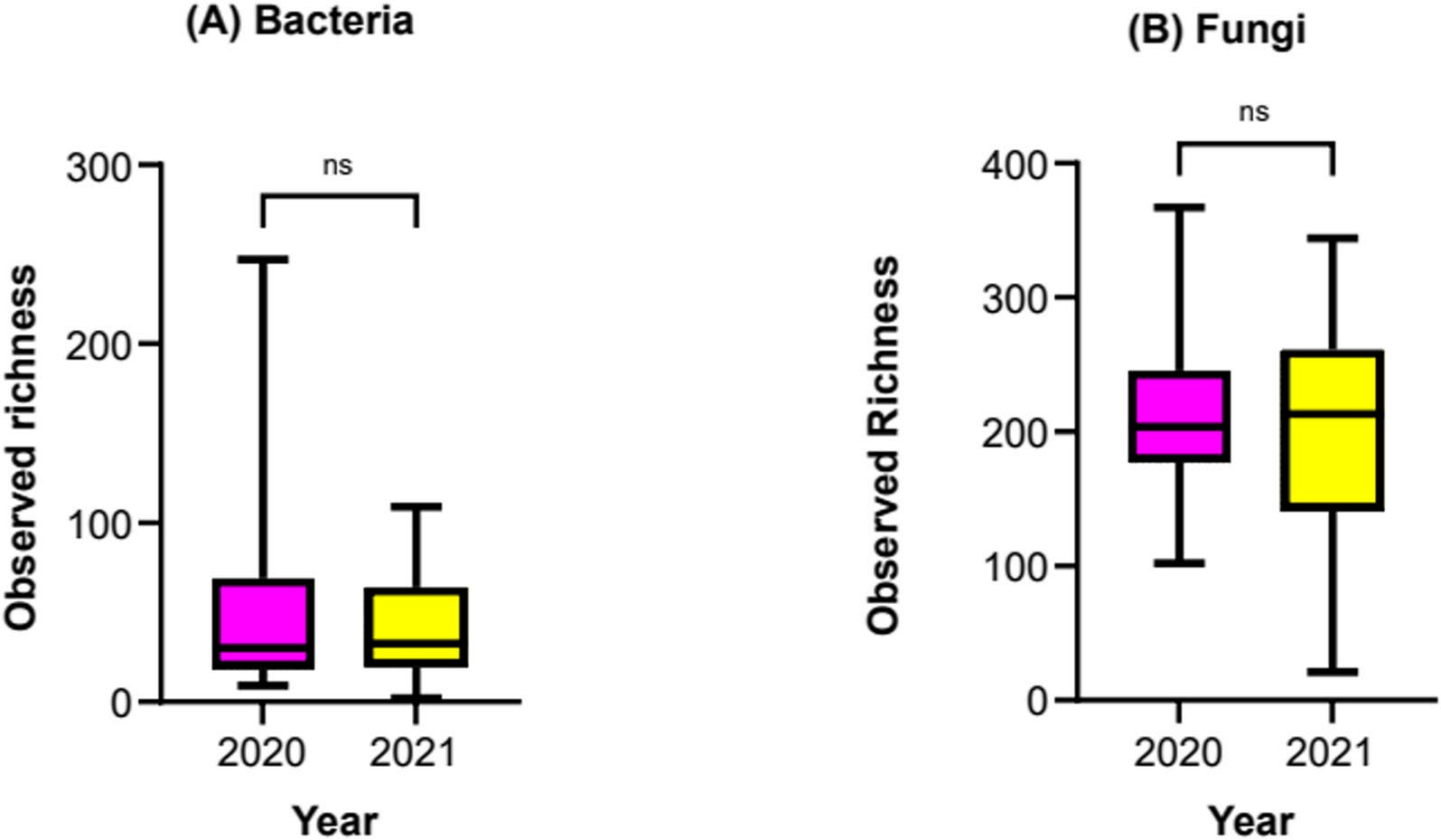
Observed richness of bacteria (left) and fungal (right) taxa present on *Pinus radiata* pollen (24 sampling sites) collected over 2 years

The observed bacterial and fungal pollen microbiome richness, across sampling years, is given in Fig. 2. Richness of fungal taxa on pollen was much greater than for bacteria, averaging over 200 (2020 = 212; 2021 = 203) ASV’s per sample compared, to less than 60 (2020 = 57; 2021 = 44) for bacteria. For both fungi and bacteria, the richness of microbiomes present on pollen was consistent across the sampling years (Wilcoxon-test; bacteria *p* = 0.81, fungi *p* = 0.96).

As total microbiome richness per sample was relatively low (i.e. tens- to hundreds of taxa per sample), we expected the MiSeq sequencing runs to capture the full extent of taxa present. Indeed, doing so can be important for increasing the accuracy of alpha-diversity estimates as well as down-stream investigation of community composition. To confirm this, rarefaction curves were generated for samples that had undergone the routine filtering mentioned above post DADA2; these are given in Figure S1. The number of unique ASV’s per sample and total reads recorded per sample for bacteria and fungi are given in Table S3 and Table S4 respectively. The dominance of plastid sequences (96.57% of total reads) (Table S2) in the sequence libraries combined with relatively low bacterial richness (Fig. 2A), resulted in the majority of 16S rRNA gene sequencing (28/33 samples) having < 4,000 tag sequence reads (Table S3). For reference, the dominant plastid sequence obtained is included in (Table S5). Conversely, more fungal sequences passed through QC (Table S1) and sequencing depth was greater, with all samples having from 16,000 to 150,000 (Table S4) useful reads for ASV generation and taxonomic assignment.

### Composition of the *Pinus radiata* pollen microbiome and defining the main taxa present

**Fig. 3:**
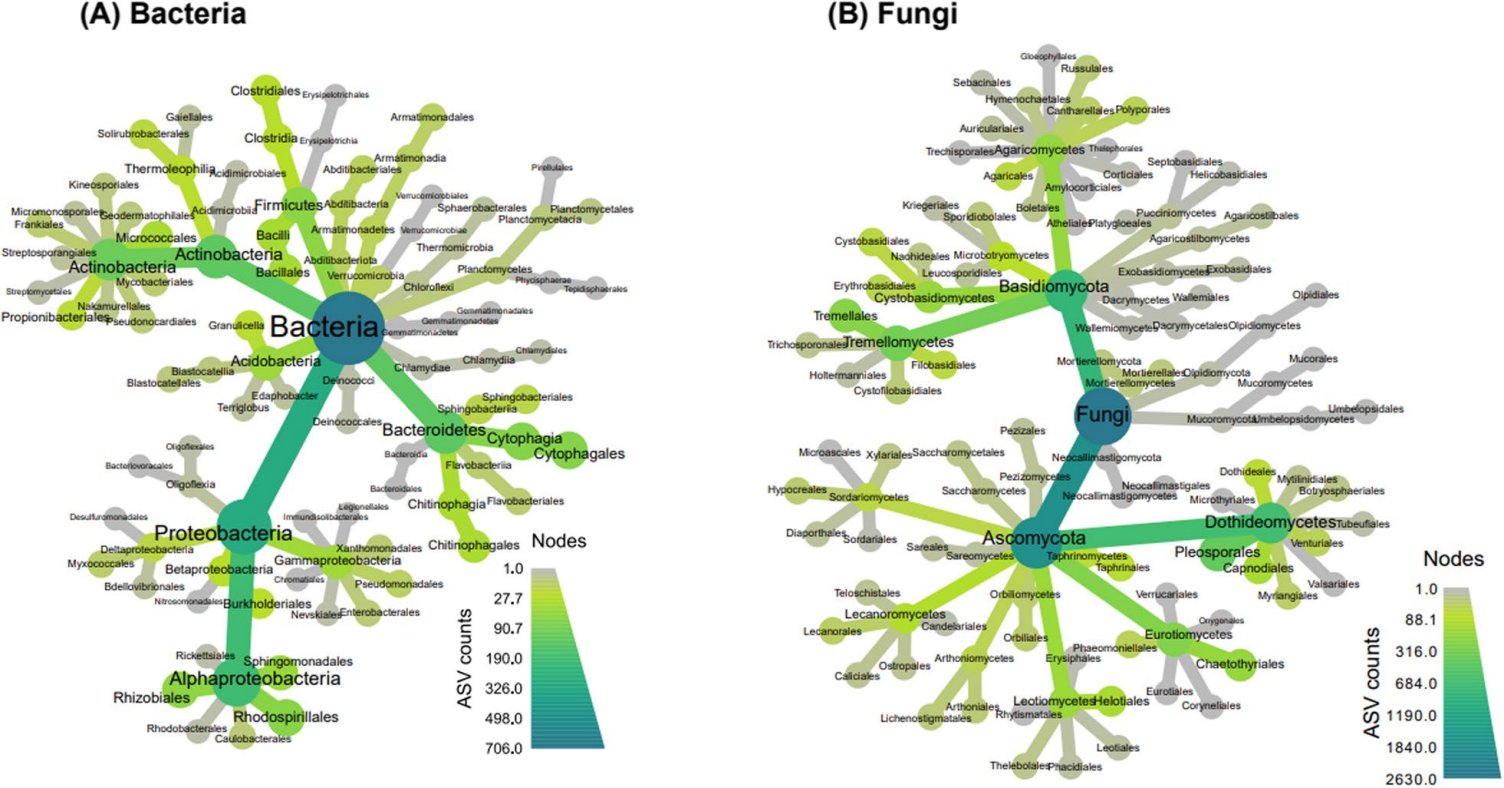
Phylogenetically hierarchical distribution (‘metacoder plots’) for bacteria (left) and fungal (right) microbiomes present on *Pinus radiata* pollen. Darker gradient fill indicates increase in ASV count of taxa on branches and leaves. Plots combine data for sites and years to indicate the overall spectrum of taxa found. Size of nodes indicate ASV counts

The microbiome of *P. radiata* pollen is dominated by fungi, having 3.7-fold greater ASV’s in comparison to bacteria. These findings are in line with measures of observed richness (i.e. Fig. 2). The fungal community was dominated by *Dothidiomycetes* (*Ascomycetes*), and *Tremellomycetes* (*Basidiomycete*). A few other sub-phyla including *Mucoromyceta*, *Mortierellomycota*, and *Eurotiomycetes* were also present, but at lower abundances (Fig. 3).

The bacterial taxa present in the pollen microbiome were more evenly distributed across the phyla and classes present than was observed for the fungal microbiome. However, trends were still evident. Within the *Proteobacteria*, *Alpha*-*proteobacteria* were numerically important. Similarly, the taxa present in the phylum *Actinobacteria* were concentrated within the class *Actinobacteria* (Fig. 3). Thus, although a greater range of phyla was present compared with fungi, the diversity within each phyla was lower.

**Fig. 4:**
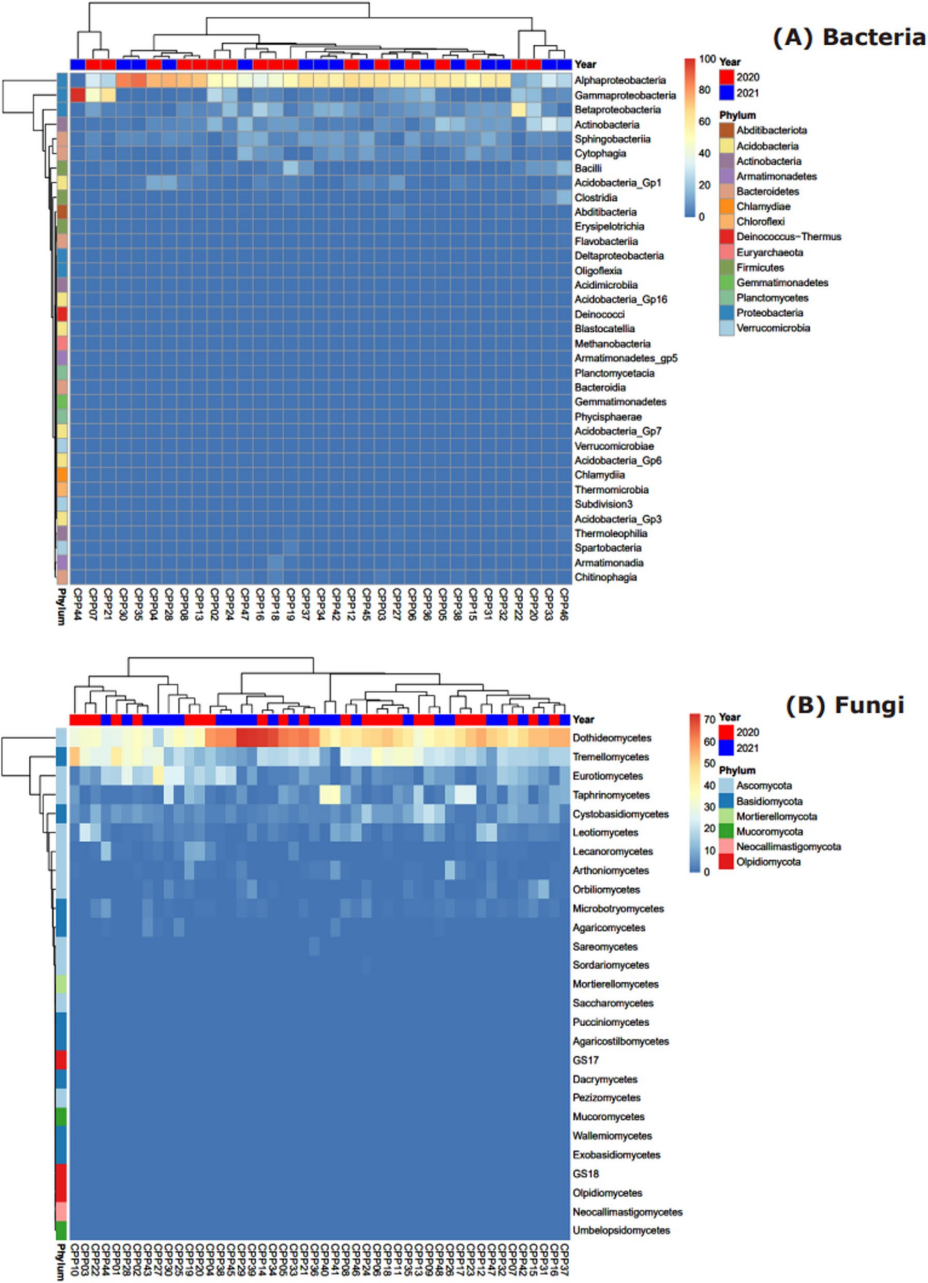
Relative abundance (%) heatmaps of bacteria (top) and fungi (bottom) *Pinus radiata* pollen microbiomes at class level. The x-axis denotes individual sample locations; coloured tiles on the top of each heatmap indicate sampling years and coloured tiles on the left indicate corresponding phylum

Data was retained at class-level aggregation for inspection of dominance and variation of taxa among samples providing some context towards the observations in Fig. 3. These are presented in Fig. 4A for bacteria, and Fig. 4B for fungi.

The bacterial community of the *P. radiata* pollen microbiome is dominated by *Proteobacteria*, particularly *Alphaproteobacteria* (Fig. 3A). The only other phyla to have numerically meaningful abundances at class level were *Actinobacteria*, *Bacteroidetes* (*Sphingobacteriia* and *Cytophagia*), *Firmicutes* (*Bacilli* and *Clostridia*), and *Acidobacteria* (GP_1). Each of these groups exhibited sample-specific variation. For example, *Betaproteobacteria* was the most abundant in the sample collected from the Glenroy 2020 collection (relative abundance > 50%), but was either absent or was only present in the range of 0.9 to 23.3% for all other samples. In contrast, *Alphaproteobacteria* was not only the most abundant amongst the samples (ranging in relative abundance from 11 to 88.3%, Fig. 4A), but was also present in nearly all samples. The only exception was a sample collected from Russel Flats in 2021; this was comprised entirely of *Gammaproteobacteria*.

The fungal community was dominated by *Ascomycota* and *Basidiomycota* taxa (Fig. 3B). *Dothiodeomycetes* were the most abundant group across samples, ranging in relative abundance from 16.9 to 72.8% of the fungi present, and next were *Tramellomycetes* (0.9 to 52.3% relative abundance, Fig. 4B). As for the bacteria, individual pollen samples exhibited anomalously high abundances of some fungal groups. For example, samples collected from Ashley Gorge in 2021 and View Hill in 2021 had 33.9% and 35.3% of *Taphrinomycetes*, respectively, but other samples had as little as 0.2% or up to 25.6%. A similar trend was also observed across classes *Eurotiomycetes* and *Cystobasidiomycetes*.

### The pine pollen metabolome

**Fig. 5:**
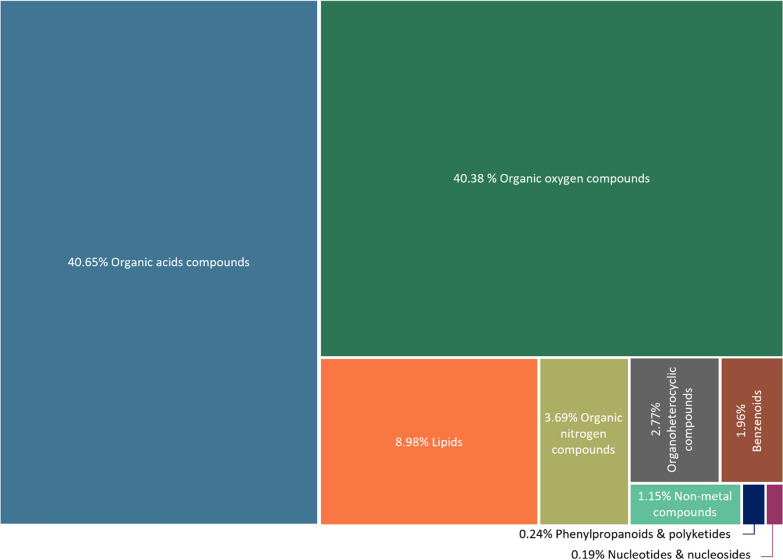
Primary metabolite compounds found on the surface of *Pinus radiata* pollen. Compounds are grouped at superclass level and the relative abundance of the metabolites are standardised as a percentage of total compounds from 12 pollen samples

The prominent metabolites present on the surface of *P. radiata* pollen are amino acids and organic oxygen compounds (primarily carbohydrates and alcohols). These comprised ~ 81% of the pine pollen metabolome (Fig. 5). At the compound level, proline was by far the most dominant metabolite, present at 17.8%. Other amino acids with notable abundances were alanine, γ-aminobutyric acid (GABA), valine, and oxoproline (1.4–3.8%). Of the organic oxygen compounds, pinitol (a cyclic polyol that's been found to be associated with other pine trees) and glycerol were both of high abundance, at ~ 5%. Fructose, glucose and sucrose were the three most prominent sugars at 2–3%. The most prominent lipids present were two prenol lipds: dehydroabietic acid (3.19%) and abietic acid (1.8%). Several phenolic compounds and flavonoids such as ρ-coumaric acid and catechin were also found, although at low concentrations (< 0.1% per compound).

### Does geographic distance or annual variation influence pollen microbiome composition?

**Fig. 6:**
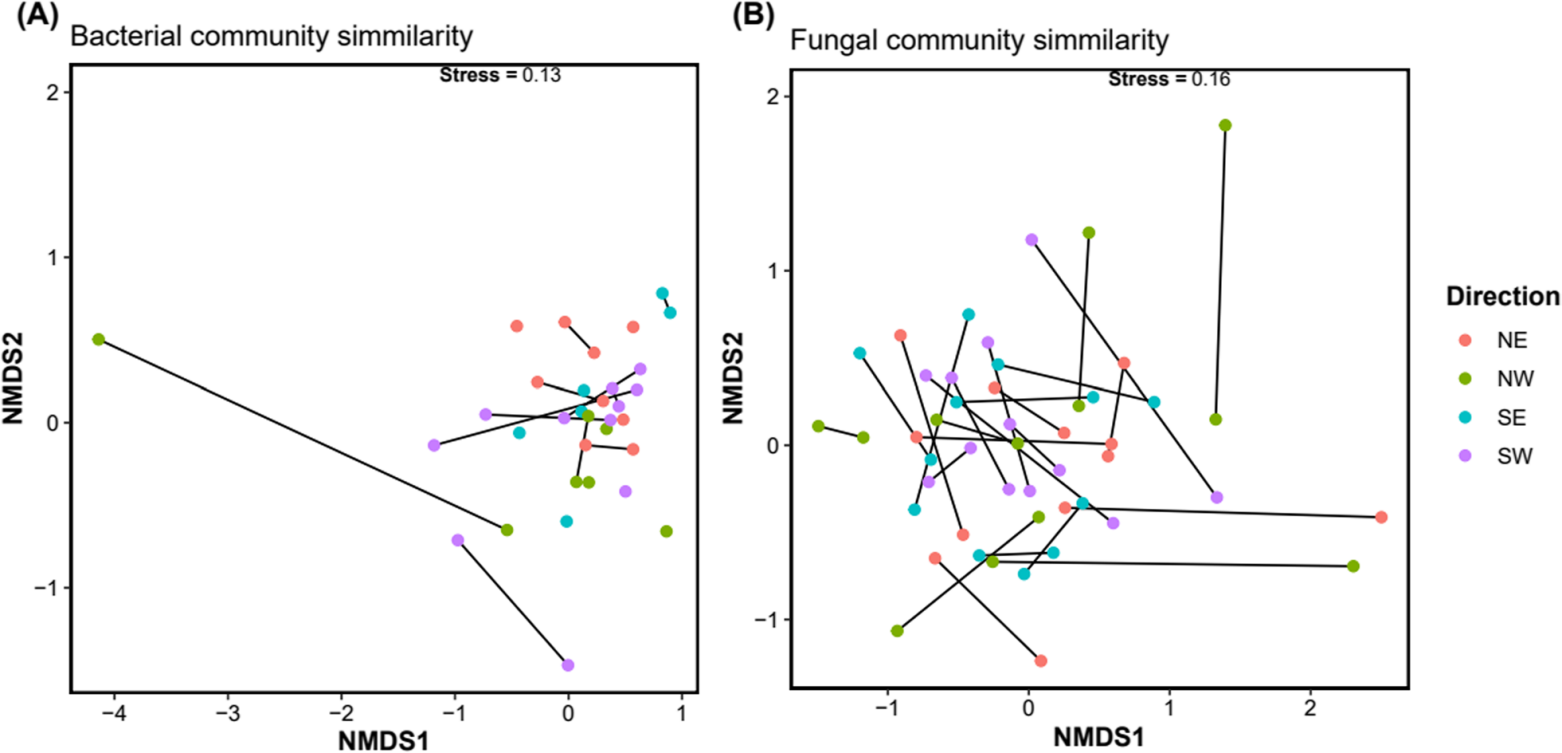
nMDS ordination plots showing similarity among samples of pollen in bacteria (**A**) and fungal (**B**) community composition by sampling year (black line) and cardinal direction location (coloured dot). Samples from the same location are connected by year of sampling. Note that some pollen samples had no bacterial microbiome in one of the sampling years; these locations therefore appear as single points

The influence of cardinal location or year of collection on pollen microbiome composition was tested using PERMANOVA; summary results are presented in Table 1. The pollen fungal community composition was stable over the two sample time points (*p* = 0.132) and with cardinal location (*p* = 0.601). No interaction effect was detected between the two factors (Table 1). Similarly, in the nMDS ordination, no structure or separation was evident related to cardinal location nor year of collection (Fig. 6). Thus, the pollen associated fungal community was stable both over time of collection and with cardinal location grouping.

**Table 1:** Summary PERMANOVA results table, testing effects of year, cardinal location and their interaction on the bacterial and fungal microbiome composition of *Pinus radiata* pollen

Taxa	Year		Cardinal direction		Year × cardinal direction	
	√CV	p_perm_	√CV	P_perm_	√CV	p_perm_
Bacteria	− 2.518	0.465	6.547	0.142	**14.944**	**0.028**
Fungi	2.987	0.132	− 1.863	0.601	2.472	0.321

Values in bold are Pperm values <0.05 and are statistally significant

√CV = square root of the component of variation associated for each term. Model residual √CV = 27.1 for bacteria and √CV = 19.0 for fungi. p_perm_ = probability statistic derived from permutation (999 x)

Similarly, bacterial community showed no variation directly related to sampling year (*p* = 0.465), nor sampling location (cardinal direction *p* = 0.142) (Table 1). However, an interaction term of year x cardinal direction was evident, and the variation component associated with this was strong (√CV = 14.944; *p* = 0.028). Exploration of this effect (pair-wise tests) suggested an influence of sampling across the SW v NW quadrants in relation to microbiome composition in 2020 (*p* = 0.059) and in 2021 (*p* = 0.074).

The grouping of samples into cardinal quadrants was used to explore the broad influence of location in the PERMANOVA based testing and apportion this variance (if any) relative to year and test for interaction effects. However, this grouping can be somewhat arbitrary and may mask secondary underlying relationship among biologic or abiotic factors associated with microbiome assembly over this sampling area. For example, samples collected at points close to the geographic centroid of the collection range could be separated into NE, SE, NW, and SW groups, yet would be effectively adjacent in absolute distance. The opposite would be true at the edge of the sampling area. As such, further analysis was conducted based on calculated pair-wise distances among sample locations to provide robust determination of the relationships between change in biological communities with geographic range.

Towards this, Mantel-based testing was used to formally test for evidence of association (rank-correlation) between the geographic and biological-based distance matrices. For the fungal community, weak correlation (ρ = 0.089) was evident. However, evidence for association between changes in bacterial community and sampling range was stronger (ρ = 0.227). Given this, only bacterial community associations were further explored.

Using BIO-ENV matching, changes in bacterial community that were associated with spatial variation were tested. Individual taxa exhibiting strongest distance-dependent relationships were *Clostridia* (ρ = 0.225), candidate division WPS-1 (ρ = 0.217), *Sphingobacteriia* (ρ = 0.213), and a non-classified *Proteobacteria* class (ncC_*Proteobacteria*; ρ = 0.184). Higher correlations were present when bacterial groups were combined; the highest included either *Sphingobacteriia* in combination with *Clostridia* (ρ = 0.275), ncC_*Proteobacteria* (ρ = 0.288) and *Alphaproteobacteria* (ρ = 0.258), andother groups. Importantly, however, *Sphingobacteriia* and *Clostridia* were both important but could often be exchanged for each other in the models indicating that, as well as being additively important (ρ = 0.275; as before), they also explained similar variance when combined with other taxa. The combination of taxa in which highest correlation with geographic distance was found included *Sphingobacteriia*, ncC_*Proteobacteria*, *Clostridia*, *Oligoflexia* and either *Acidimicrobiia* or *Deinococci* (ρ = 0.346, p = 0.005). Relationships between change in taxa abundances and geographic sampling are given in Fig. S2.

### The core microbiome of *Pinus radiata* pollen

Core genera were defined as those having a minimum 50% prevalence and 0.01 detection threshold across samples [33, 39]. Detection threshold is the minimum relative abundance (i.e. 1%) value at which groups are considered present in the community while prevalence of 50% indicates that groups must be present in at least 50% of the samples to be considered a core member. The core microbiome conserved across all samples (locations and years) are summarised in Table 2. Overall, fungi had twice as many core genera compared to bacteria. An UpSet plot showing core genera between and across years and highlighting subtle sampling time-based influences are shown in Figure S3.

**Table 2:** The core bacterial and fungal genera, prevalent across at least 50% of the samples at a minimum of 0.01 detection threshold. The relative abundance ranges from the lowest abundance in a sample to the highest abundance in a sample

	Relative abundance %	Phylogenetic lineage
Bacteria		
* Robbsia*	0.85–23.26%	*Proteobacteria; Betaproteobacteria; Burkholderiales; Burkholderiaceae*
*Sphingomonas*	2.22–23.23%	*Proteobacteria; Alphaproteobacteria; Sphingomonadales; Sphingomonadaceae*
*Hymenobacter*	0.45–12.55%	*Bacteroidota; Cytophagia; Cytophagales; Hymenobacteraceae*
*Acidisoma*	0.63–5.13%	*Proteobacteria; Alphaproteobacteria; Rhodospirillales; Acetobacteraceae*
*Methylobacterium*	0.3–5.79%	*Proteobacteria; Alphaproteobacteria; Hyphomicrobiales; Methylobactericeae*
Fungi		
*Microsphaeropsis*	0.1–46.24%	*Ascomycota; Dothideomycetes; Plenosporales; Didymosphaeriaceae*
*Perusta*	0.12–44.26%	*Ascomycota; Pezizomycotina; Dothideomycetes; Dothideomycetidae; Dothideomycetidae incertae sedis*
*Hormonema*	0.12–34.01%	*Ascomycota; Pezizomycotina; Dothideomycetes; Dothideomycetidae; Dothideales; Dothioraceae*
*Taphrina*	0.14–30.98%	*Ascomycota; Taphrinomycotina; Taphrinomycetes; Taphrinales; Taphrinaceae*
*Gelidatrema*	0.02–24.76%	*Basidiomycota; Agaricomycotina; Tremellomycetes; Tremellales; Phaeotremellaceae*
*Naganishia*	0.06–10.28%	*Basidiomycota; Agaricomycotina; Tremellomycetes; Filobasidiales; Filobasidiaceae*
*Vishniacozyma*	0.13–10.32%	*Basidiomycota; Agaricomycotina; Tremellomycetes; Tremellales; Bulleribasidiaceae*
*Genolevuria*	0.08–8.66%	*Basidiomycota; Agaricomycotina; Tremellomycetes; Tremellales; Bulleraceae*
*Epicoccum*	0.21–8.36%	*Ascomycota; Dothideomycetes; Pleosporales; Didymellaceae*
*Symmetrospora*	0.17–4.88%	*Basidiomycota; Pucciniomycotina; Cystobasidiomycetes; Cystobasidiomycetes incertae sedis; Symmetrosporaceae*

## Discussion

### Pine pollen hosts a fungal rich microbiome

Our results demonstrated that *Pinus radiata* pollen does host a microbiome, and this microbiome is an order-of-magnitude richer in fungal taxa compared with bacteria. This finding differs to currently reported angiosperm species where the pollen is typically richer in bacterial taxa. Manirajan et al*.* [52], for example, investigated pollen grains from 8 species: birch/*Betula*, canola/*Brassica*, rye/*Secale*, autumn crocus/*Colchicum*, common hazel/*Corylus*, blackthorn/*Prunus*, common mugwort/*Artemisia*, and cherry plum/*Prunus*. Each individual plant species had a characteristic pollen microbiome and, among these, differences between wind and insect-pollenated species were then present. Across these plant species, the pollen’s bacteria and fungi had a similar number of OTUs and the bacteria was more diverse than the fungi. Similar findings were observed in the works of [59] when looking at the pollen of birch (*Betula*) and timothy grass (*Phleum*).

The finding that fungal rich pine-pollen microbiome differs to that of angiosperm species is consistent with the literature, in that different plant species, including gymnosperms like *Pinus radiata*, host distinct pollen microbiomes. It should also be noted, however, that richness does not equate to more abundance (sensu biomass) of a particular group. For example, while the overall number of fungal taxa on *P. radiata* pollen maybe greater than bacteria, the total relative population size might be considerably smaller. Fungal dominance maybe in species present, not total abundance per se.

A unique attribute of pine pollen is the durable exterior coating (exine) made of sporopollenin. Sporopollenin is one of the most chemically inert biological polymers known and helps ensure pollen is chemically resistant and environmentally durable [77]. Each pollen exine has a unique and complex surface topology and chemistry enabling specificity with stigmas of reproductively compatible flowers. Immediately below the exine is an ‘intine’ inner later. Intine is composed of cellulose and hemi-cellulose, pectin and other proteins, and callose, and supports and regulates the growth of the pollen tube during fertilization. In some respects, pine pollen should comprise a preferred habitat for fungal colonisation. For example, the hydrophobicity properties of the exine layer (also pectin within the pollen) can favour fungal groups possessing surface-active hydrophobin proteins, allowing for attachment onto hydrophobic and hydrophilic surfaces [79, 87], such as pollen. Furthermore, antimicrobial peptides are present on surfaces of some pollen [22]. These are hypothesised to play a role in preventing self-pollination of angiosperms [57], but may also constrain the diversity of bacteria present on pollen surfaces [88]. These factors combined could initially be considered to facilitate colonisation of fungal groups over bacterial groups in this unique microhabitat. However, many of these are properties typical of all pollen—not just those from conifers. Furthermore, and as noted previously, most studies describe a greater richness in bacterial species on pollen. As such, additional or alternative explanations need to be considered. These could be, for example: (1) strong host-level filtering based on selective chemicals, such as peptides noted by Zasloff [88] but likely including other groups, (2) the pollen microbiome is a reflection of the catkin microbiome or other microbiomes present on the pine which is, in itself, fungal dominated and contributes taxa to the pollen; (3) the environmental microbiome is a dominant contributor onto pollen and unique properties of conifer-forest ecosystems that result in fungal dominance are reflected in the assembly of microbiomes on pollen.

Pine pollen is a known useful source of nutrients such as carbohydrates, proteins, lipids, phenolics, and minerals, which are essential for the wider ecosystem microbiota [32, 63]. It is also used as nutrient supplements in human health [12]. With this in mind, it is not surprising to find that pollen is a suitable habitat for growth of a multitude of bacteria and fungi. Whilst there has been considerable research on the chemical composition of pine pollen, the nutrients present vary depending on the pine species itself as well as environmental factors such as altitude and soil characteristics [12]. Moreover, the process of undertaking chemical analysis can be destructive and, as such, the final data set often includes intracellular nutrients/metabolome as well. Given this, it is not always evident what metabolites are surficial on pollen and available for the microbes inhabiting this habitat.

Our analysis found that the *P. radiata* pollen surface metabolome contained a high percentage of amino acids and sugars, both of which are important nutrient and energy resources for microbial growth. Of particular note was proline being, by far, the most abundant metabolite present. Other pine pollens also exhibit significantly high levels of proline, including *Pinus jeffreyi* (Jeffrey pine) and *Pinus contorta* (Lodgepole pine), [7]. The accumulation of proline in plants has been observed as a reaction to environmental stresses, including exposure to UV radiation [71, 78]. As pollen is transported by the wind, it becomes exposed to UV radiation. The presence of proline may serve as a protective mechanism for the haploid genome within the pollen.

There were also several flavonoids and polyphenols present and these are known to have antimicrobial properties that shape microbial communities on plant phyllospheres. Specifically, ρ-coumaric acid was the most abundant phenolic compound, which is a known antibacterial agent [60], the presence of compounds like this could result in fewer bacteria present on the pollen. At this stage, however, this is purely speculative. Dehydroabietic acid (terpenoid) is found in resin and woody material of pine species. It has a broad range of metabolic and biological properties, including antimicrobial activity [35]. While a major constituent of resins and associated biomass burning and commercial lumber processing mills [45], dehydroabietic acid is rarely reported in pollen. A notable exception is Jeffrey pine pollen [7]. In addition to potential implications for microbiome selection/filtering on pollen, the presence of dehydroabietic acid on pollen needs to be considered in terms of its interaction with hydroxyl radicals and subsequent influence on atmospheric chemistry [7].

### Geographic range and time are secondary influences of microbiome assembly

The environment is a natural and important source of microbiomes for plant tissues [75]. Indeed, there is ongoing bi-directional exchange of the microbiome between plants and the environment such that the systems are, in effect, an intimately interconnected meta-holobiont [81]. However, whilst this is apparent for tissues such leaves and roots [75], the connection between the pollen and environment isn’t as well defined. The work of Obersteiner et al. [59] demonstrated that even on different plant species occurring in the same location, the microbiome is most likely originating from the host plant,the environment was a second-order driver, with plant host-specific factors being most important. Similarly, Manirajan et al. [54] assessed the pollen microbiome of plant species situated in and across different geographic areas and found pollen microbiomes were primarily host-specific. These studies indicate that the pollen microbiome assembly is primarily influenced by factors associated with the host plant.

We explored if different sampling locations influenced assembly of pine pollen microbiome. In this study, *P. radiata* was collected within a 2500 km^2^ sampling area and included trees in general proximity to a wide range of land uses. We initially grouped samples to cardinal locations relative the centre point of sampling [as noted earlier, these groupings were subjectively assigned and only used for exploratory analysis]. Sampling location/environment had no direct influence on the bacterial nor fungal microbiome on *P. radiata* pollen. We further tested to see if geographic distances among sampling locations was related to microbiome assembly. For example, in our sampling region the eastern areas are coastal and have a drier and warmer climate than those close to the mountainous (further west). In this analysis, effects of sample location could be determined, but were weak. Importantly, the dominant part of the pine pollen microbiome (i.e. the fungi), exhibited stability or conservation in community structure irrespective of sampling location. No change in geographic-related sampling was evident. Rather, it was components of the bacterial community in which changes in the abundance of some taxa occurred in association with sampling distance.

*Clostridia* was the most notable taxa to change in abundance over space, exhibiting a roughly west-to-east gradient in distribution (from high to low abundance, respectively). *Sphingobacteriia* was the most abundant species and was present in nearly all samples, whereas *Acidimicrobiia*, *Deinococci* and *Oligoflexia* were only present in 1–3 samples with no obvious correlation. In these instances, we hypothesise that environmental filtering of specific bacterial taxa may be occurring, where perhaps factors related to tree health or other stochastic influences within the environment are influencing colonisation onto tree tissues. We observe the legacy of this within the pollen microbiome simply as a surface where microbes from the environment are deposited.

Likewise, there were no significant differences in fungal and bacterial community composition across the two annual time points. However, with regards to bacteria, some distinctions emerged. In 2020, certain classes such as *Verrucomicrobiae*, *Thermomicrobia*, *Phycisphaerae*, *Oligoflexia*, *Acidiimicrobia*, and *Acidobacteria* Groups 3, 6, and 7 were exclusively detected, while classes including *Methanobacteria*, *Gemmatimonadetes*, and *Bacteroidia* were only identified in 2021. Some changes to the microbiome are expected, whether arising from random fluctuations or variations in sampling from 1 year to the next. It is notable, however, that like the results for the geographic distance testing, that changes over years were observed in the bacterial and not fungal component of the microbiome. This provides additional support for the bacterial microbiome being of secondary significance to the overall pollen microbiome; its assembly isn’t as tightly regulated or consistent as that for fungi. The pine bacterial microbiome is under more stochastically influence by environmental site or sampling time than that of the fungal community. It's important to note that the sampling methods remained consistent between the two time points, and the timing of the sample collection was the same. However, factors like the pollen release from catkins, seasonal changes and weather conditions are all variable. Overall, it is evident that neither sampling year nor geographic distance are the main dominant drivers of the pine pollen microbiome, particularly the dominant fungal community component which demonstrated remarkable stability.

### Is the microsporangiate a primary source of the pollen microbiome?

Examination of the *P. radiata* catkins and pollen by SEM revealed that catkins had visibly rich microbial growth on its surface. Relative to the catkins, pollen grains themselves exhibited relatively sparse microbial presence (Suppl. Fig S4). Pollen is formed within, and released from, the male microsporangiate strobili. Microsporangia within the pollen cones produce microspores through meiosis and these develop into pollen grains. As such, the formation of pollen occurs within the enclosed cones, being isolated from the external environment until the cones mature and open for pollen release. Accordingly, the opportunity for external colonisation of pollen with microbiome is limited; microbiomes present on pollen are likely to be sourced from the surrounding catkin material. It is likely, therefore, that movement of microbiomes from the catkin to the pollen is an important primary source of the pollen microbiome. Indeed, there has been a long history of use of bleach treatment of catkins to reduce mould and other growth on pine pollen [80], further demonstrating fungi present on pollen originates from surface of the catkins. If validated, host influence of the pollen microbiome may occur via properties related to the chemistry and ecology of the catkin structure itself, and focus on the metabolome and ecology of this tissue may shed light on processes affecting assembly and transmission of the pollen microbiome.

Not all the microbiome may reside on the surface of pollen. Following sterilisation, growth of endophytic microbiota, such as *Enterobacter cloacae*, has been demonstrated in a range of *Pinus* spp [50]. The functional significance of these isolates is unknown, however strong co-evolutionary processes typically drive plant-endophytic associations [43, 48] and, as such, shared fitness outcomes are likely for both the plant and microorganism.

### Pine pollen has a core microbiome

There was a consistent presence of specific fungal and bacterial taxa across *P. radiata* pollen samples; this indicates that pine pollen holds symbiosis with a core microbiome. This core microbiome is, axiomatically, the stable fraction being consistently present (see below) over 2 years of sampling and across locations spanning broad geographic range and proximity to land uses. Indeed, the term ‘core microbiome’ is described as one that hosts anywhere from 30 to 95% of microbial taxa [68] across samples, depending on the habitat being investigated. In our study, we define a core member as having a 50% prevalence and 0.01% relative abundance detection threshold [33, 39]. From these parameters, five bacterial and ten fungal core genera were identified. At a higher taxonomic level such as phyla, the dominant bacterial and fungal phyla on radiata pollen was *Proteobacteria* and *Ascomycota*, which are also dominant on pollen for a range of wind and insect pollinating plants [52]. At the genera level, *Robbsia, Sphingomonas, Hymenobacter, Acidisoma* and *Methylobacterium* were the dominant genera of the core bacterium community. Of these, *Robbsia* and *Methylobacterium* have been found on birch and canola pollen previously [52, 76], and *Sphingomonas* are well documented as a core bacterium in pollen collected by bees [19]. *Methylobacterium* are a genus of well-documented phyllosphere epiphytes that secrete cytokinins and auxin plant hormones [70]. *Hymenobacter* and *Acidisoma* have not yet been reported to be associated with pollen, but are known plant phyllosphere bacteria [6, 67].

The core fungal genera were *Genolevuria, Microsphaeropsis, Gelidatrema, Epicoccum, Perusta, Taphrina, Vishniacozyma, Hormonema, Naganishia*, and *Symmetrospora*. The fungal families *Didymosphaeriaceae* and *Dothioraceae*, *Taphrinaceae* were also found in the 8 pollen species studied by Manirajan et al. [52], with *Dothioraceae* being prominent throughout most of the pollen species. This aligns with our study of pine pollen too, where *Dothideomycetes* was the most abundant class of fungi present.

The presence of *Dothideomycetes* is not surprising as it contains a wide range of plant-associated fungi with ecological roles ranging from saprophytes, pathogens, to endophytes [73]. It is also the dominant fungal group on *P. radiata* needles [1, 2]. Not surprisingly there were more fungal core groups compared to bacterial groups, which is expected due to the sheer number of fungal groups recorded compared to bacterial groups. These results support those of Manirajan et al. [52] who report more core fungi than bacteria on pollen from eight plant species, despite these pollens all having greater bacterial richness.

Comparing our results to the core microbiomes from the species of pollen Manirajan et al. [52] studied, of which there were 33 fungal core genera and 12 bacterial core genera, only *Vishniacozyma* and *Taphrina* fungi were shared with these other pollen species. This shows that the pollen microbiome is indeed species specific, giving rise to unique groups especially at finer taxonomic resolution such as genera. As before, this provides support that the host plant is key in supplying/recruiting or maintaining specific and stable microbiome community. At a higher taxonomic level such as phyla, taxonomic lineages of both bacteria and fungi in this study (i.e. dominance of *Proteobacteria* and *Ascomycota*) appear to be consistent across other pollen microbiome studies. It is also not surprising, therefore, that the pollen microbiome of a gymnosperm species, such as *P. radiata*, might be considerably different to that of angiosperm plant species described to date.

### Methodological considerations

The presence of plant organelle DNA (e.g. chloroplast and mitochondrial) can be a problematic and an unwanted component of 16S rRNA generated libraries in plant microbiome research. In some regards these plant organelles are part of the ‘phytobiome’ per se, extending our understanding of host associations across the continuum from opportunistic/casual, through to enriched (e.g. rhizosphere), to tissue colonising, endophytic and ancestral endosymbionts. In this study however, the DNA from these ancient cellular symbionts were unwanted.

Although there are no chloroplasts in pollen, pollen does contain non-photosynthetic plastids which populated the 16S rRNA libraries of this study; these were accounted for using deep sequencing followed by bioinformatic subtraction of the unwanted plastid reads. We ensured there was sufficient sequencing depth to cover the 16S rRNA sequences of the pollen microbiome, and this was confirmed by inspection of rarefaction curves which had surpassed asymptote. This approach is the most commonly used in plant microbiome research, providing an unbiased and comprehensive view of the microbiome.

We have previously tested pPNA and mPNA-based plant blockers [47] for 16S rRNA gene based microbiome characterisation of *Pinus radiata* material [1, 2]. The use of these reduced plastid origin sequences on NGS libraries by approximately 23%. However, they also introduced strong sequences biases, significantly altering community composition relative to reference samples. Furthermore, given the blockers were not 100% effective, bioinformatic identification and removal of plastid origin sequences are still required. As potential benefits were outweighed by biases introduced into the sequencing, we avoid these for *P. radiata* microbiome research [1, 2]. Furthermore, we also evaluated different 16S rRNA primer combinations. While these reduced chloroplast amplification, we found an increase in mitochondrial DNA amplification. These issues are widely recognised [31]. In short, the primers described in our study (515F and 806R) combined with deep sequencing and bioinformatic filtering of subsequent NGS libraries offers a balanced approach to capturing microbial diversity on and in *P. radiata* plant tissues.

### Limitations and future research

Due to the experimental system used, this study was conducted on field-collected pollen specimens from *P. radiata*. Control of tree genotype was not conducted, nor age of trees measured. However, all likely originated from commercial stock of *P. radiata* domesticated from parent material originally sourced from California via Australia [8]. Given the findings reaffirm the seminal role of the host (as opposed to the environment) in shaping pollen microbiome communities, further work should consider inclusion of host genotype in their design (i.e. in situations where host genotypes likely vary). Further to this, locations in which trees were sampled ranged from road-side shelterbelts to small stands of trees, and to individual specimens in recreational parks. These factors were not (and could not be) formally tested within the analysis, yet possibly contribute to some of the total variation captured in the ‘environmental’ testing. However, as this was found to be of limited importance in microbiome assembly we propose that further investigation of this may not be generally needed (e.g. structured sampling of pines based on adjacent land use) for general pine microbiome characterisation. Finally, only visibly heathy trees were chosen for this study. Thus, influence of stress from factors such as drought, disease, pollution etc. could not be assessed, but may be important [59]. These should be considered in future research.

The domestication and economic importance of the species means that a wealth of information continues to be generated on its wider biology, including physiology and genetics. Robust systems are in place for propagation, transformation and other experimentation on *Pinus radiata*, and a wide network of existing trials are in place to test aspects of the tree's phenotype and fitness in diverse environments [8]. Similarly, an increasing wealth of information is being built on the microbiome of *P. radiata* including needles, roots, wood, and rhizosphere [1, 2, 65, 69]. The results presented here have shown the importance of considering the pollen microbiome when examining the overall microbiome of trees. Further research investigating whether this microbiome is passed down vertically to seeds and seedlings will be important. Moreover, exploring whether the influx of microbes each spring plays a role in shaping the phyllosphere, litter layer, or soil microbiomes, as well as in the dissemination of pathogens. This work will go towards answering key questions related to the assembly and function of the *P. radiata* tree-microbiome system, improving the fitness and resilience of forests in a rapidly changing world.

## Supplementary Information

Additional file 1.

## Data Availability

Sequencing data from this project can be found on the Short Read Archive (SRA) database, under the BioProject accession number PRJNA1109979. GitHub link for all scripts used during DADA2 processing, filtering post DADA2 and for figure generation in R can be found here https://github.com/syaliny/Pine-pollen-Microbiome.git.

## References

[1] Addison S, Armstrong C, Wigley K, Hartley R, Wakelin S. What matters most? Assessment of within-canopy factors influencing the needle microbiome of the model conifer, Pinus radiata. Environ Microbiome. 2023;18(1):45. 10.1186/s40793-023-00507-8. 37254222 10.1186/s40793-023-00507-8 PMC10230745

[2] Addison S, Rúa MA, Smaill SJ, Daley KJ, Singh BK, Wakelin SA. Getting to the root of tree soil microbiome sampling. Phytobiomes. 2023. 10.1094/PBIOMES-09-22-0060-R.

[3] Addison SL, Rúa MA, Smaill SJ, Singh BK, Wakelin SA. Partner or perish: tree microbiomes and climate change. Trends Plant Sci. 2024;29(9):1029–40. 10.1016/j.tplants.2024.03.008. 38641475 10.1016/j.tplants.2024.03.008

[4] Agarwal VK, Sinclair JB. Principles of seed pathology. 2nd ed. Boca Raton: CRC Press; 1997.

[5] Anderson MJ. A new method for non-parametric multivariate analysis of variance. Austral Ecol. 2001;26(1):32–46. 10.1111/j.1442-9993.2001.01070.pp.x.

[6] Ares A, Pereira J, Garcia E, Costa J, Tiago I. The leaf bacterial microbiota of female and male kiwifruit plants in distinct seasons: assessing the impact of Pseudomonas syringae pv. actinidiae. Phytobiomes J. 2021;5(3):275–87. 10.1094/pbiomes-09-20-0070-r.

[7] Axelrod K, Samburova V, Khlystov AY. Relative abundance of saccharides, free amino acids, and other compounds in specific pollen species for source profiling of atmospheric aerosol. Sci Total Environ. 2021;799: 149254. 10.1016/j.scitotenv.2021.149254. 34375869 10.1016/j.scitotenv.2021.149254

[8] Burdon R, Libby W, Brown A. Domestication of radiata pine. Cham: Springer; 2017. 10.1007/978-3-319-65018-0.

[9] Callahan BJ, McMurdie PJ, Rosen MJ, Han AW, Johnson AJ, Holmes SP. DADA2: high-resolution sample inference from Illumina amplicon data. Nat Methods. 2016;13(7):581–3. 10.1038/nmeth.3869. 27214047 10.1038/nmeth.3869 PMC4927377

[10] Caporaso JG, Lauber CL, Walters WA, Berg-Lyons D, Huntley J, Fierer N, et al. Ultra-high-throughput microbial community analysis on the Illumina HiSeq and MiSeq platforms. ISME J. 2012;6(8):1621–4. 10.1038/ismej.2012.8. 22402401 10.1038/ismej.2012.8 PMC3400413

[11] Cellini A, Giacomuzzi V, Donati I, Farneti B, Rodriguez-Estrada MT, Savioli S, et al. Pathogen-induced changes in floral scent may increase honeybee-mediated dispersal of Erwinia amylovora. ISME J. 2019;13(4):847–59. 10.1038/s41396-018-0319-2. 30504898 10.1038/s41396-018-0319-2 PMC6461938

[12] Cheng Y, Wang Z, Quan W, Xue C, Qu T, Wang T, et al. Pine pollen: a review of its chemical composition, health effects, processing, and food applications. Trends Food Sci Technol. 2023;138:599–614. 10.1016/j.tifs.2023.07.004.

[13] Clarke K, Ainsworth M. A method of linking multivariate community structure to environmental variables. Mar Ecol Prog Ser. 1993;92:205–19. 10.3354/meps092205.

[14] Clarke KR. Non-parametric multivariate analyses of changes in community structure. Aust J Ecol. 1993;18(1):117–43. 10.1111/j.1442-9993.1993.tb00438.x.

[15] Cole JR, Wang Q, Fish JA, Chai B, McGarrell DM, Sun Y, et al. Ribosomal Database Project: data and tools for high throughput rRNA analysis. Nucleic Acids Res. 2014;42(Database issue):D633–42. 10.1093/nar/gkt1244. 24288368 10.1093/nar/gkt1244 PMC3965039

[16] Conti I, Medrzycki P, Argenti C, Meloni M, Vecchione V, Boi M, et al. Sugar and protein content in different monofloral pollens—building a database. Bull Insectol. 2016;69:318–20.

[17] Conway J, Lex A, Gehlenborg N. UpSetR: an R package for the visualization of intersecting sets and their properties. Bioinformatics 2017;33:2938-2940. 10.1093/bioinformatics/btx364 10.1093/bioinformatics/btx364 PMC5870712 28645171

[18] Cullen NP, Fetters AM, Ashman TL. Integrating microbes into pollination. Curr Opin Insect Sci. 2021;44:48–54. 10.1016/j.cois.2020.11.002. 33248285 10.1016/j.cois.2020.11.002

[19] Dew RM, McFrederick QS, Rehan SM. Diverse diets with consistent core microbiome in wild bee pollen provisions. Insects. 2020. 10.3390/insects11080499. 32759653 10.3390/insects11080499 PMC7469187

[20] Dharampal PS, Carlson C, Currie CR, Steffan SA. Pollen-borne microbes shape bee fitness. Proc Biol Sci. 2019;286(1904):20182894. 10.1098/rspb.2018.2894. 31185869 10.1098/rspb.2018.2894 PMC6571465

[21] Donati I, Cellini A, Buriani G, Mauri S, Kay C, Tacconi G, et al. Pathways of flower infection and pollen-mediated dispersion of Pseudomonas syringae pv. actinidiae, the causal agent of kiwifruit bacterial canker. Hortic Res. 2018;5:56. 10.1038/s41438-018-0058-6. 30393538 10.1038/s41438-018-0058-6 PMC6210195

[22] Doughty J, Dixon S, Hiscock SJ, Willis AC, Parkin IA, Dickinson HG. PCP-A1, a defensin-like Brassica pollen coat protein that binds the S locus glycoprotein, is the product of gametophytic gene expression. Plant Cell. 1998;10(8):1333–47. 10.1105/tpc.10.8.1333. 9707533 10.1105/tpc.10.8.1333 PMC144068

[23] Ersts PJ. Geographic Distance Matrix Generator (version 1.2.3). American Museum of Natural History, Centre for Biodiversity and Conservation. https://biodiversityinformatics.amnh.org/open_source/gdmg/. Accessed 08 Sep 2023.

[24] FAO. Global planted forests thematic study: results and analysis bADL, J. Ball and J. Carle. Planted forests and trees working paper, 38. Rome, Italy, 2006.

[25] FAO. Forests and genetically modified trees. Rome: FAO; 2010.

[26] FAO W, IFAD. The state of food insecurity in the world 2012. Economic growth is necessary but not sufficient to accelerate reduction of hunger and malnutrition. Rome, Italy, 2012. https://www.fao.org/3/i3027e/i3027e.pdf. 10.3945/an.112.003343 PMC3648734 23319131

[27] Fielding JM. Branching and flowering characteristics of Monterey Pine. Forest Timber Bureau Bull. 1960;37:38-39.

[28] Filipiak M. Pollen stoichiometry may influence detrital terrestrial and aquatic food webs. Front Ecol Evol. 2016;4:138. 10.3389/fevo.2016.00138.

[29] FOA. Facts & figures 2016/17 New Zealand plantation forest industry. Wellington, New Zealand. 2016. https://www.nzfoa.org.nz/images/Facts_Figures_2019_20_Web_FA3-updated.pdf

[30] Foster ZS, Sharpton TJ, Grünwald NJ. Metacoder: an R package for visualization and manipulation of community taxonomic diversity data. PLoS Comput Biol. 2017;13(2):e1005404. 10.1371/journal.pcbi.1005404. 28222096 10.1371/journal.pcbi.1005404 PMC5340466

[31] Giangacomo C, Mohseni M, Kovar L, Wallace JG. Comparing DNA extraction and 16S rRNA gene amplification methods for plant-associated bacterial communities. Phytobiomes J. 2020;5(2):190–201. 10.1094/PBIOMES-07-20-0055-R.

[32] Graham MD, Vinebrooke RD, Turner M. Coupling of boreal forests and lakes: effects of conifer pollen on littoral communities. Limnol Oceanogr. 2006;51(3):1524–9. 10.4319/lo.2006.51.3.1524.

[33] Graystock P, Rehan SM, McFrederick QS. Hunting for healthy microbiomes: determining the core microbiomes of Ceratina, Megalopta, and Apis bees and how they associate with microbes in bee collected pollen. Conserv Genet. 2017;18(3):701–11. 10.1007/s10592-017-0937-7.

[34] Greenfield L. Plant pollen production in selected tree species. Canterb Bot Soc J. 1996;31:10–3.

[35] Hao M, Xu J, Wen H, Du J, Zhang S, Lv M, et al. Recent advances on biological activities and structural modifications of dehydroabietic acid. Toxins (Basel). 2022. 10.3390/toxins14090632. 36136570 10.3390/toxins14090632 PMC9501862

[36] Hutchison LJ, Barron GL. Parasitism of pollen as a nutritional source for lignicolous Basidiomycota and other fungi. Mycol Res. 1997;101(2):191–4. 10.1017/S095375629600233X.

[37] Ihrmark K, Bodeker IT, Cruz-Martinez K, Friberg H, Kubartova A, Schenck J, et al. New primers to amplify the fungal ITS2 region–evaluation by 454-sequencing of artificial and natural communities. FEMS Microbiol Ecol. 2012;82(3):666–77. 10.1111/j.1574-6941.2012.01437.x. 22738186 10.1111/j.1574-6941.2012.01437.x

[38] Illumina. bcl2fastq Conversion Software. Illumina, Inc.; 2019. https://support.illumina.com/sequencing/sequencing_software/bcl2fastq-conversion-software.html.

[39] Kardas E, González-Rosario AM, Giray T, Ackerman JD, Godoy-Vitorino F. Gut microbiota variation of a tropical oil-collecting bee species far exceeds that of the honeybee. Front Microbiol. 2023;14:1122489. 10.3389/fmicb.2023.1122489. 37266018 10.3389/fmicb.2023.1122489 PMC10229882

[40] Kim BR, Shin J, Guevarra R, Lee JH, Kim DW, Seol KH, et al. Deciphering diversity indices for a better understanding of microbial communities. J Microbiol Biotechnol. 2017;27(12):2089–93. 10.4014/jmb.1709.09027. 29032640 10.4014/jmb.1709.09027

[41] Kolde R. pheatmap: Pretty Heatmaps; 2012. https://CRAN.R-project.org/package=pheatmap.

[42] Kõljalg U, Larsson KH, Abarenkov K, Nilsson RH, Alexander IJ, Eberhardt U, et al. UNITE: a database providing web-based methods for the molecular identification of ectomycorrhizal fungi. New Phytol. 2005;166(3):1063–8. 10.1111/j.1469-8137.2005.01376.x. 15869663 10.1111/j.1469-8137.2005.01376.x

[43] Koskella B, Bergelson J. The study of host-microbiome (co)evolution across levels of selection. Philos Trans R Soc Lond B Biol Sci. 1808;2020(375):20190604. 10.1098/rstb.2019.0604. 10.1098/rstb.2019.0604 PMC7435161 32772660

[44] Lahti L, Shetty S, Ernst F, Borman T, Braccia DJ, Huang R, et al. Tools for microbiome analysis in R, 2017.

[45] Lai C, Liu Y, Ma J, Ma Q, He H. Laboratory study on OH-initiated degradation kinetics of dehydroabietic acid. Phys Chem Chem Phys. 2015;17(16):10953–62. 10.1039/C5CP00268K. 25824374 10.1039/c5cp00268k

[46] López-Orozco R, García-Mozo H, Oteros J, Galán C. Long-term trends and influence of climate and land-use changes on pollen profiles of a Mediterranean oak forest. Sci Total Environ. 2023;897:165400. 10.1016/j.scitotenv.2023.165400. 37423282 10.1016/j.scitotenv.2023.165400

[47] Lundberg DS, Yourstone S, Mieczkowski P, Jones CD, Dangl JL. Practical innovations for high-throughput amplicon sequencing. Nat Methods. 2013;10(10):999–1002. 10.1038/nmeth.2634. 23995388 10.1038/nmeth.2634

[48] Lyu D, Zajonc J, Pagé A, Tanney CAS, Shah A, Monjezi N, et al. Plant holobiont theory: the phytomicrobiome plays a central role in evolution and success. Microorganisms. 2021. 10.3390/microorganisms9040675. 33805166 10.3390/microorganisms9040675 PMC8064057

[49] Ma H, Crowther TW, Mo L, Maynard DS, Renner SS, van den Hoogen J, et al. The global biogeography of tree leaf form and habit. Nat Plants. 2023;9(11):1795–809. 10.1038/s41477-023-01543-5. 37872262 10.1038/s41477-023-01543-5 PMC10654052

[50] Madmony A, Chernin L, Pleban S, Peleg E, Riov J. Enterobacter cloacae, an obligatory endophyte of pollen grains of Mediterranean pines. Folia Microbiol (Praha). 2005;50(3):209–16. 10.1007/BF02931568. 16295659 10.1007/BF02931568

[51] Manirajan BA, Hinrichs AK, Ratering S, Rusch V, Schwiertz A, Geissler-Plaum R, et al. Bacterial species associated with highly allergenic plant pollen yield a high level of endotoxins and induce chemokine and cytokine release from human A549 cells. Inflammation. 2022;45(6):2186–201. 10.1007/s10753-022-01684-3. 35668156 10.1007/s10753-022-01684-3 PMC9646606

[52] Manirajan BA, Maisinger C, Ratering S, Rusch V, Schwiertz A, Cardinale M, et al. Diversity, specificity, co-occurrence and hub taxa of the bacterial-fungal pollen microbiome. FEMS Microbiol Ecol. 2018. 10.1093/femsec/fiy112. 29878113 10.1093/femsec/fiy112

[53] Manirajan BA, Ratering S, Cardinale M, Maisinger C, Schnell S, Heidt PJ, et al. The microbiome of flower pollen and its potential impact on pollen-related allergies; 2019.

[54] Manirajan BA, Ratering S, Rusch V, Schwiertz A, Geissler-Plaum R, Cardinale M, et al. Bacterial microbiota associated with flower pollen is influenced by pollination type, and shows a high degree of diversity and species-specificity. Environ Microbiol. 2016;18(12):5161–74. 10.1111/1462-2920.13524. 27612299 10.1111/1462-2920.13524

[55] Martin M. Cutadapt removes adapter sequences from high-throughput sequencing reads. EBMnet.journal. 2011;17(1):10–2. 10.14806/ej.17.1.200.

[56] McMurdie PJ, Holmes S. phyloseq: an R package for reproducible interactive analysis and graphics of microbiome census data. PLoS ONE. 2013;8(4):e61217. 10.1371/journal.pone.0061217. 23630581 10.1371/journal.pone.0061217 PMC3632530

[57] Nasrallah JB. Recognition and rejection of self in plant reproduction. Science. 2002;296(5566):305–8. 10.1126/science.296.5566.305. 11951033 10.1126/science.296.5566.305

[58] Nelson EB. The seed microbiome: origins, interactions, and impacts. Plant Soil. 2018;422(1):7–34. 10.1007/s11104-017-3289-7.

[59] Obersteiner A, Gilles S, Frank U, Beck I, Haring F, Ernst D, et al. Pollen-associated microbiome correlates with pollution parameters and the allergenicity of pollen. PLoS ONE. 2016;11(2):e0149545. 10.1371/journal.pone.0149545. 26910418 10.1371/journal.pone.0149545 PMC4765992

[60] Ojha D, Patil KN. p-Coumaric acid inhibits the Listeria monocytogenes RecA protein functions and SOS response: An antimicrobial target. Biochem Biophys Res Commun 2019;517(4):655-661. 10.1016/j.bbrc.2019.07.093 10.1016/j.bbrc.2019.07.093 31416617

[61] Oksanen J, Simpson, G, Blanchet F, Kindt R, Legendre P, Minchin P, et al. vegan: Community Ecology Package_. R package In., version 2.6-4 edn; 2022.

[62] Parada AE, Needham DM, Fuhrman JA. Every base matters: assessing small subunit rRNA primers for marine microbiomes with mock communities, time series and global field samples. Environ Microbiol. 2016;18(5):1403–14. 10.1111/1462-2920.13023. 26271760 10.1111/1462-2920.13023

[63] Pawlik MM, Ficek D. Spatial distribution of pine pollen grains concentrations as a source of biologically active substances in surface waters of the Southern Baltic Sea. Water. 2023;15(5):978.

[64] Payn T, Harnett M, Scott G. Radiata pine pollen info sheet; 2017.

[65] Prihatini I, Glen M, Wardlaw TJ, Mohammed CL. Diversity and identification of fungi associated with needles of Pinus radiata in Tasmania. South For J For Sci. 2016;78(1):19–34. 10.2989/20702620.2015.1092345.

[66] R Core Team. R: a language and environment for statistical computing. Vienna, Austria: R Foundation for Statistical Computing; 2021.

[67] Reis VM, Teixeira KR. Nitrogen fixing bacteria in the family Acetobacteraceae and their role in agriculture. J Basic Microbiol. 2015;55(8):931–49. 10.1002/jobm.201400898. 25736602 10.1002/jobm.201400898 PMC7166518

[68] Risely A. Applying the core microbiome to understand host-microbe systems. J Anim Ecol. 2020;89(7):1549–58. 10.1111/1365-2656.13229. 32248522 10.1111/1365-2656.13229

[69] Rúa MA, Wilson EC, Steele S, Munters AR, Hoeksema JD, Frank AC. Associations between ectomycorrhizal fungi and bacterial needle endophytes in Pinus radiata: implications for biotic selection of microbial communities. Front Microbiol. 2016;7:399. 10.3389/fmicb.2016.00399. 27065966 10.3389/fmicb.2016.00399 PMC4815291

[70] Sanjenbam P, Shivaprasad PV, Agashe D. Impact of phyllosphere methylobacterium on host rice landraces. Microbiol Spectr. 2022;10(4):e0081022. 10.1128/spectrum.00810-22. 35856668 10.1128/spectrum.00810-22 PMC9431194

[71] Saradhi PP, Alia AS, Prasad KV. Proline accumulates in plants exposed to UV radiation and protects them against UV induced peroxidation. Biochem Biophys Res Commun. 1995;209(1):1–5. 10.1006/bbrc.1995.1461. 7726821 10.1006/bbrc.1995.1461

[72] Schermer É, Bel-Venner M-C, Gaillard J-M, Dray S, Boulanger V, Le Roncé I, et al. Flower phenology as a disruptor of the fruiting dynamics in temperate oak species. New Phytol. 2020;225(3):1181–92. 10.1111/nph.16224. 31569273 10.1111/nph.16224

[73] Schoch C, Grube M. Pezizomycotina: Dothideomycetes and Arthoniomycetes. In: McLaughlin DJ, Spatafora JW, editors. Systematics and evolution: part B. Berlin: Springer; 2015. p. 143–76.

[74] Schwendemann AB, Wang G, Mertz ML, McWilliams RT, Thatcher SL, Osborn JM. Aerodynamics of saccate pollen and its implications for wind pollination. Am J Bot. 2007;94(8):1371–81. 10.3732/ajb.94.8.1371. 21636505 10.3732/ajb.94.8.1371

[75] Sessitsch A, Wakelin S, Schloter M, Maguin E, Cernava T, Champomier-Verges MC, et al. Microbiome interconnectedness throughout environments with major consequences for healthy people and a healthy planet. Microbiol Mol Biol Rev. 2023;87(3):e0021222. 10.1128/mmbr.00212-22. 37367231 10.1128/mmbr.00212-22 PMC10521359

[76] Shi H, Manirajan BA, Ratering S, Geissler-Plaum R, Schnell S. Robbsia betulipollinis sp. nov., isolated from pollen of birch (Betula pendula). Curr Microbiol. 2023;80(7):234. 10.1007/s00284-023-03344-7. 37278851 10.1007/s00284-023-03344-7 PMC10244264

[77] Stanley RG, Linskens HF. Pollen biology biochemistry management. 1st ed. Berlin: Springer; 1974.

[78] Szabados L, Savouré A. Proline: a multifunctional amino acid. Trends Plant Sci. 2010;15(2):89–97. 10.1016/j.tplants.2009.11.009. 20036181 10.1016/j.tplants.2009.11.009

[79] Takahashi T, Maeda H, Yoneda S, Ohtaki S, Yamagata Y, Hasegawa F, et al. The fungal hydrophobin RolA recruits polyesterase and laterally moves on hydrophobic surfaces. Mol Microbiol. 2005;57(6):1780–96. 10.1111/j.1365-2958.2005.04803.x. 16135240 10.1111/j.1365-2958.2005.04803.x

[80] Tulecke WR. Preservation and germination of the pollen of Ginkgo under sterile conditions. Bull Torrey Bot Club. 1954;81:509.

[81] Vannier N, Mony C, Bittebiere AK, Michon-Coudouel S, Biget M, Vandenkoornhuyse P. A microorganisms’ journey between plant generations. Microbiome. 2018;6(1):79. 10.1186/s40168-018-0459-7. 29695286 10.1186/s40168-018-0459-7 PMC5918900

[82] Vorholt JA. Microbial life in the phyllosphere. Nat Rev Microbiol. 2012;10(12):828–40. 10.1038/nrmicro2910. 23154261 10.1038/nrmicro2910

[83] Wardle DA, Bardgett RD, Klironomos JN, Setala H, van der Putten WH, Wall DH. Ecological linkages between aboveground and belowground biota. Science. 2004;304(5677):1629–33. 10.1126/science.1094875. 15192218 10.1126/science.1094875

[84] Wassermann B, Abdelfattah A, Wicaksono WA, Kusstatscher P, Muller H, Cernava T, et al. The Brassica napus seed microbiota is cultivar-specific and transmitted via paternal breeding lines. Microb Biotechnol. 2022;15(9):2379–90. 10.1111/1751-7915.14077. 35593114 10.1111/1751-7915.14077 PMC9437892

[85] White T, Bruns T, Lee S, Taylor J, Innis M, Gelfand D, et al. Amplification and direct sequencing of fungal ribosomal RNA genes for phylogenetics; 1990, pp. 315–22.

[86] Wickham H. ggplot2: elegant graphics for data analysis; 2016.

[87] Wosten HA, Schuren FH, Wessels JG. Interfacial self-assembly of a hydrophobin into an amphipathic protein membrane mediates fungal attachment to hydrophobic surfaces. EMBO J. 1994;13(24):5848–54. 10.1002/j.1460-2075.1994.tb06929.x. 7813424 10.1002/j.1460-2075.1994.tb06929.x PMC395559

[88] Zasloff M. Pollen has a microbiome: implications for plant reproduction, insect pollination and human allergies. Environ Microbiol. 2017;19(1):1–2. 10.1111/1462-2920.13661. 28097828 10.1111/1462-2920.13661

